# Lateral flow immunoassay for small-molecules detection in phytoproducts: a review

**DOI:** 10.1007/s11418-022-01605-6

**Published:** 2022-02-16

**Authors:** Poomraphie Nuntawong, Waraporn Putalun, Hiroyuki Tanaka, Satoshi Morimoto, Seiichi Sakamoto

**Affiliations:** 1 Graduate School of Pharmaceutical Sciences, Kyushu University, 3-1-1 Maidashi, Higashi-ku, Fukuoka, 812-8582 Japan; 2 Faculty of Pharmaceutical Sciences, Khon Kaen University, Khon Kaen, 40002 Thailand; 3 School of Pharmacy, Sanyo-Onoda City University, 1-1-1 Daigakudouri, Sanyo-onoda-shi, Yamaguchi, 756-0884 Japan; 4 Research Group for Pharmaceutical Activities of Natural Products Using Pharmaceutical Biotechnology (PANPB), National Research University-Khon Kaen, Khon Kaen, Thailand

**Keywords:** Immunoassay, Immunochromatographic strip test, Lateral flow immunoassay, Plant secondary metabolites, Hapten, Small molecules

## Abstract

Phytoproducts are involved in various fields of industry. Small-molecule (Mw < 900 Da) organic compounds can be used to indicate the quality of plant samples in the perspective of efficacy by measuring the necessary secondary metabolites and in the perspective of safety by measuring the adulterant level of toxic compounds. The development of reliable detection methods for these compounds in such a complicated matrix is challenging. The lateral flow immunoassay (LFA) is one of the immunoassays well-known for its simplicity, portability, and rapidity. In this review, the general principle, components, format, and application of the LFA for phytoproducts are discussed.

## Introduction

Phytoproducts are the substances, extracts, or compounds obtained from plants. They are in high demand in several industries, including medicine, cosmetics, and foods [1]. Plant secondary metabolites are an important part of phytoproducts [2]. The secondary metabolites of plants are generally small-molecule organic compounds (Mw < 900 Da) produced by plants but not directly involved in their growth and development [1, 3, 4]. The secondary metabolites are produced for long-term plant survival against herbivores, pests, pathogens, and the attraction of pollinators. The role of certain secondary metabolites remains unclear [1, 3–5]. These compounds have various biological functions, which can be applied in many fields [2]. The quality of phytoproducts is important, particularly when the plants are used in the field of medicine. The level of secondary metabolites is typically measured and used as a quality indicator for phytoproducts [6, 7]. When plant-based products were used in a particular field, the adulterants and contaminants were highlighted as a global problem [7–11]. Certain products have been spiked with fungicides and/or phytoregulators for agricultural purposes [12]. The excessive intake of these adulterated and contaminated phytoproducts is harmful. Thus, a series of analytical techniques for small-molecules detection such as high-performance liquid chromatography (HPLC), gas chromatography (GC), GC-mass spectrometry detection (GC–MS), and liquid chromatography-mass spectrometry (LC–MS) have been developed. However, these methods require sophisticated equipment, skillful operators, and long operation times. Furthermore, these methods cannot be applied outside the laboratory.

The concept of using immunoassays for small-molecule detection was introduced to surmount these limitations. The assays are based on the specific binding of an antibody and antigen. The enzyme-linked immunosorbent assay (ELISA) relies on this basis. The benefits of this method are its cost-effectiveness, simplicity, and sufficient sensitivity, indicating that this immunological approach is useful for secondary metabolite detection [13]. Although the ELISA can solve the drawbacks of conventional chromatographic assays, the competitive ELISA is not suitable for certain scenarios and requires improvements. Generally, indirect competitive ELISA (icELISA) comprises five main steps, namely the antigen coating, nonspecific binding blocking, primary antibody reaction, enzyme-labeled antibody reaction, and enzymatic reaction. Hence, at least 4.5 h is required to complete the general icELISA [13]. Moreover, to read the signal from ELISA, microplate readers that correspond to the signal are required. Given these pitfalls in the current detection systems, the point-of-care test called a lateral flow immunoassay (LFA) was developed. Generally, macromolecules (proteins) or whole cells are the major analytes detected by immunoassays. These analytes can bind to the solid phase (i.e., microplate for ELISA and membrane for LFA) with few or without modification. Thus, non-competitive immunoassays are possible for detection. Moreover, their large and complex structure enables the generation of two different antibodies which recognize different epitopes on the same antigen with ease. This enables sandwich immunoassay format possible when macromolecules and whole cells are used as analytes. The competitive format also can be developed as an optional method for macromolecules and whole cell immunoassays. For small-molecules detection, the immunoassay format and design are slightly different. The competitive format is preferred over non-competitive format since the small molecules are unable to be immobilized directly on the solid phase. The general fabrication of LFA and its application in quality control for phytoproducts are discussed in this paper.

### LFA

There is a wide application of point-of-care tests in environmental science, food, drug, and clinical analyses. “Ready to read” results are provided in a short time. The LFA or immunochromatographic strip test is a point-of-care device, which has been applied in qualitative, semi-quantitative, and quantitative analyses in versatile scenarios for six decades. The LFA is a simplified immunoassay, in which the antibody–reporter molecule conjugates are accumulated on the designated area of the membrane, which is filled with the antigen, and the result can be read-out within several minutes.

In 1956, the first nanoparticle application in immunoassays was initiated during Plotz and Singer’s so-called “latex fixation” test, in which the immunological approach was developed without using a paper-based device [14]. Thereafter, the radioimmunoassay (RIA) was developed by Yalow and Berson in 1959 [15], where a paraffin paper-based immunochromatographic device was developed to determine the insulin level in plasma. Afterward, the enzyme-based immunoassay was popularized as the replacement of the RIA in the 1960s [16]. The basic idea of immunoassays was continuously refined until the concept of using colloidal gold nanoparticle conjugates in an immunoassay was initiated in 1980 when the strategy called the sol-particle immunoassay was reported [17]. The usage of colloidal gold nanoparticles in immunoassays gained considerable attention in the 1980s because the well-known pregnancy test strip was commercially available and patented [18]. Eventually, the LFA was developed for determining antigens, antibodies, and amplification products of genes [19–22] in several fields. This method is suitable as a point-of-care test, according to the World Health Organization using the criteria of ASSURED (affordable, sensitive, specific, user-friendly, robust, equipment-free, and deliverable) [23]. The criteria were recently revised as REASSURED (real-time connectivity, ease of specimen collection, affordable, sensitive, specific, user-friendly, rapid and robust, equipment-free and environmentally friendly, and deliverable to end-users) [24]. The system has been continuously refined and simplified, enabling non-skilled operators to perform the assay. Various commercially available strip tests for various antigens or antibodies have been launched [25–27]. Although the classical chromatography and ELISA methods offer high accuracy, the LFA is advantageous over them in scenarios where laboratory equipment is not available. Moreover, the assay time is extremely short. This renders the LFA a premium choice for sample screening.

### Components of LFA

**Fig. 1:**
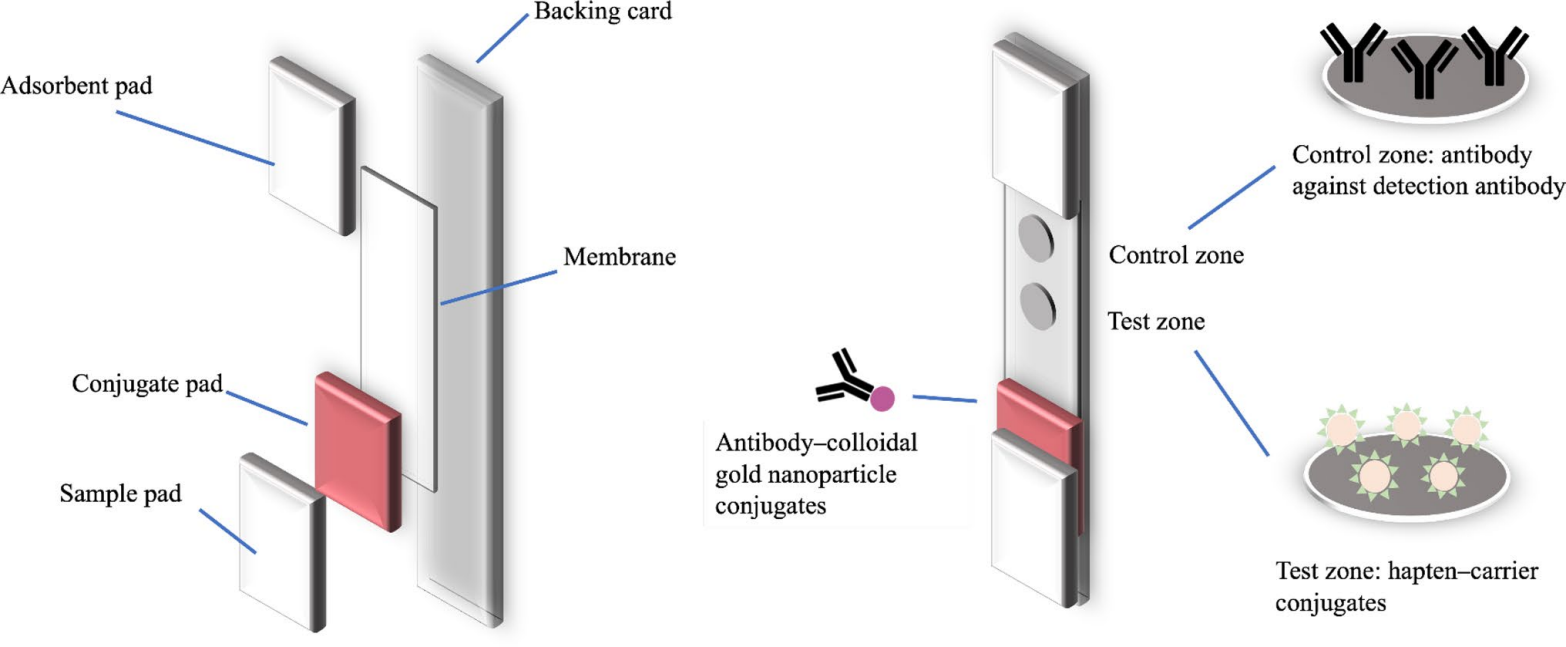
General components of the LFA and schematic description of each component of the immunochromatographic strip

Generally, as demonstrated in Fig. 1, the immunoassay is performed on a strip on which many types of materials are assembled. The main components of the strip are described in this section.

#### Backing card

This is the part that provides strength to the whole system and enables ease of handling. It is typically fabricated with plastic polymers, such as polyvinyl chloride [28, 29] and polystyrene [30]. Herein, the thickness of the backing card was in a wide range of 0.3–0.6 mm in the form of a piece of plastic sheet or a roll of plastic sheet. The commercially available backing card for LFA typically comes with an adhesive layer, which can be divided into four main areas for attaching the necessary components to the strip.

#### Sample pad

This is the component where the sample is applied to initiate the assay. The liquid is transferred through this component to other parts of the strip. The sample pad is typically made from cellulose [31], whereas certain minor pads are made from glass fiber [32]. The ideal sample pad continuously transfers the liquid at the designed flow rate. Hence, the homogeneity of the pore size is designed by the specific assay. For instance, samples that do not contain particle or prefiltered could use a homogeneous pore size sample pad, compared with samples that contain coarse materials, such as plant powders, preparations with insoluble ingredients, and whole-cell suspensions that prefer a nonhomogeneous pore size pad for the initial filter effect. When necessary, the sample pad is pretreated with a wetting agent, buffer, protein, or viscosity-enhancing agent. The aim of these pretreatments was mainly to control the flow rate, increase the retention time on the conjugate pad, and increase the reaction time at test and control zones.

#### Conjugate pad

Glass fiber is the famous material used for this component. Minor conjugate pads are made from polyester, cellulose, and other materials. It is the space where the antibody–reporter molecule conjugates are accumulated. The ideal conjugate pad should act as a bank of antibody–reporter molecule conjugates, which readily release once the liquid passes through and not permanently retain the antibody–reporter molecule conjugates. In certain LFA formats, this component is omitted, and the antibody–reporter molecule conjugates are directly added into the sample [33, 34].

#### Membrane 

This is the area where the reaction of the antibody and antigen occurs. This membrane is typically made from nitrocellulose [35]. However, certain strip membranes are made of individually customized cellulose paper [36]. The pore size of the membrane highly affects the sensitivity of the LFA through the capillary flow rate. The reaction time of the antibody and immobilized antigen is high when the flow rate is low, offering a high chance of a reaction. Nevertheless, the experiment time (time until read-out) is equally high in such cases. Therefore, the membrane is chosen based on the balance between sensitivity and assay time. The ideal membrane exhibits low nonspecific adsorption. However, membrane blocking with low protein concentration (e.g., bovine serum albumin (BSA)) can be conducted when necessary. Before use, the membrane can be modified using a wetting agent and buffer to achieve the best condition for assays.

#### Adsorbent pad

This is the area to retain excess liquid in the system. The drainage of the excess liquid reduces the backflow of the sample and maintains an even flow rate in the system. Cellulose is typically used as a material for this pad.

The specificity and sensitivity are the important features of the LFA. The specificity is the antibody-related property. However, sensitivity could be affected by components of the LFA. The sensitivity of the test is related to the flow rate of the whole assay. Thus, the components that affect the flow rate of the test, i.e., sample pad and membrane play a role on sensitivity controlling. The flow rate of the whole assay could be controlled by the materials pore size, hydrophilicity of the materials, and pretreatment of the materials. Increasing/decreasing the flow rate for sensitivity adjustment is dependent on the format of the assay. For sandwich format, the flow rate needs to be slow enough to obtain the sufficient reaction time. In the competitive format, faster flow rate is not an obstruction for this LFA since the presence of an analyte is evaluated by the absence of the spot. However, the flow rate for competitive assay should be optimized to make the test zone visible in the non-analyte control strips. Even though sensitivity is the important factor of the assay, some LFAs require fast flow rate since the analysis time is also the point of concern as LFA is usually used as rapid test.

### Manufacturing process of LFA

To date, the LFA applied for plant secondary metabolite is not available for mass-production. The mass-production of the LFA is generally applied for the medical diagnosis where the analytes are macromolecules. However, the manufacturing process of LFA is quite similar to the general one. The production of LFA generally initiated from preparation of necessary elements, assembling, cutting, and cassette assembling. The manufacturing process can be a batch-to-batch production or a continuously reel-to-reel production.

#### Membrane preparation

The test zone solution (hapten–carrier solution) and control zone solution (antibody against detection antibody) are accurately dispensed to the membrane using the appropriate dispenser, e.g., contact tip dispenser, noncontact pump-driven solenoid dispensers, and quantitative airbrush-type dispensers. The suitable dispenser is selected by the scale of the production. The reaction line-dispensed membrane is then dried using either in-line drying or batch oven drying. If necessary, the membrane is blocked by the appropriate solution using dipping tank and finally dried prior assemble. The membrane could be prepared with or without backing card in this step.

#### Conjugate pad preparation

The conjugate pad is pretreated (if necessary) with appropriate solution and dried prior to the antibody–reporter molecule conjugates dispensing. The conjugate could be dispensed by either dipping tank or the accurate dispenser. The finished conjugate pad is dried prior assembling.

#### Sample pad preparation

In some case, the LFA performance is enhanced by sample pad pretreatment. Thus, the sample pad is pretreated with optimized sample pad treatment solution and is then dried prior assemble.

#### Assembling or lamination

This is the process that membrane, conjugate pad, and sample pad are assembled into one-piece. Generally, semi-automated laminator is applied in this step in batch-to-batch production while in-line laminator which can laminate the treated elements after treatment is applied for reel-to-reel production.

#### Strip cutting

This is the process which the laminated sheet is cut into each strip. The size of the strip varies depending on the design of the strip test. The general cutters for this process are single rotary blade cutter, rotary card cutter, and guillotine cutter.

#### Cassette assembly

To increase the ease of handle for the strip, the strip is manually placed into the plastic housing. In some cases, the strip is cover with the soft plastic to enhance the strength of the strip and protect the reaction area against the mechanical force.

### General principle of the LFA

#### Competitive format

**Fig. 2:**
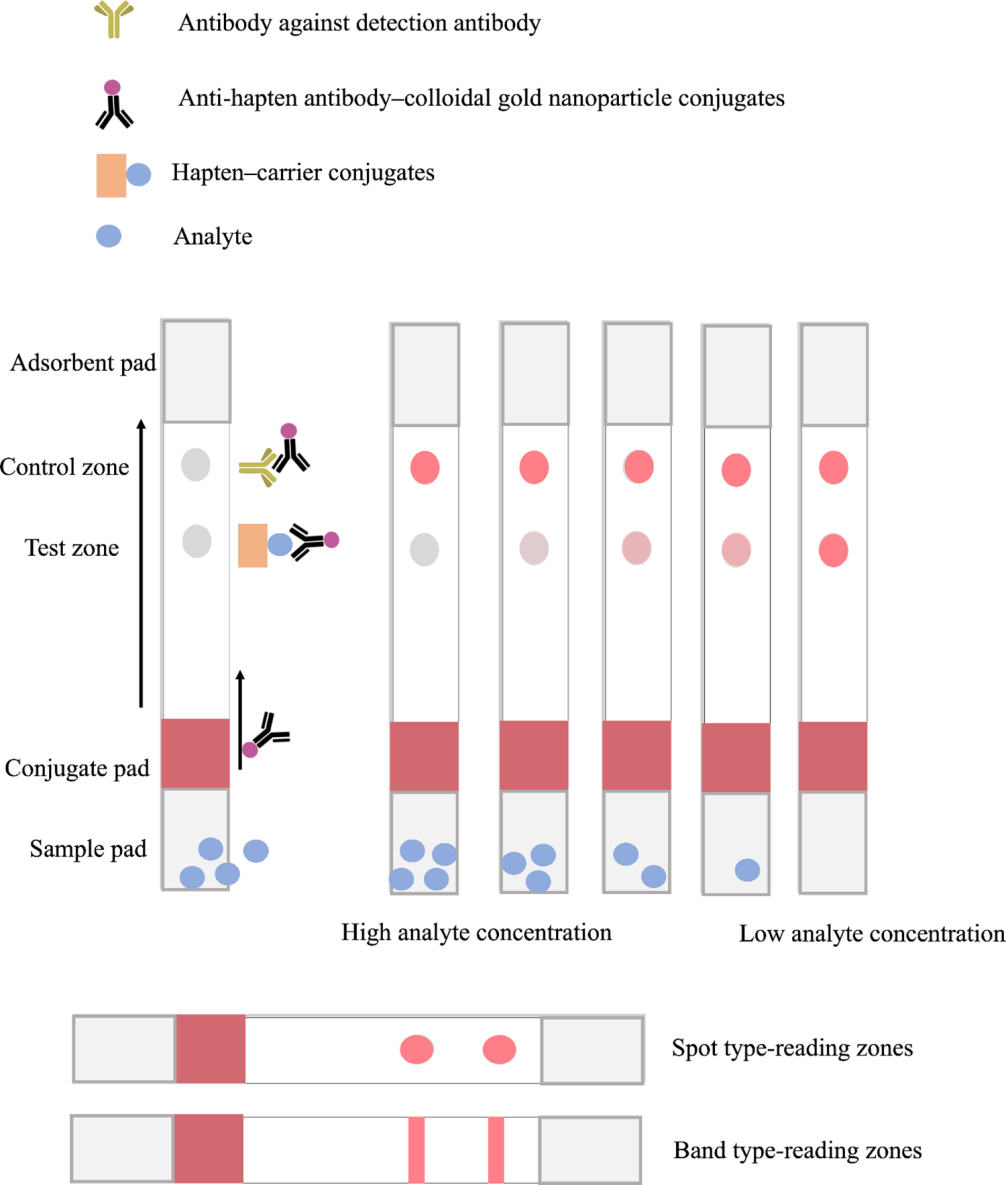
Competitive format of the strip test. The upper section demonstrates the symbolic representation of each compartment. The figure demonstrates the LFA signal in various analyte concentrations. There are two main types of detection zones, which are the spot and band types which demonstrated in the lower section

There is a simple rationale behind the LFA, as demonstrated in Fig. 2. The major assays for small-molecule detection in phytoproducts are based on the competitive immunoassay. The assay commences when the liquid containing the analyte of interest flows through the strip after sample application. The sets of the strip can be provided with the cassette, which contains the sample loading opening that is connected to the sample pad and read-out opening [37–43]. However, the cassette is not required in certain assays. Hence, the sample is applied either into the sample loading opening of the cassette or by directly dipping the sample pad of the strip into the sample solution. The liquid flows through the strip mainly by capillary force [44]. The direction of the flow is the origin of the assay name since the liquid flows laterally through the other compartment when the cassette is provided. In a case where the cassette is absent, the strip typically stands in the glass tube [33] or the microplate [34] where the sample solution or suspension is located. Therefore, the direction of the flow is antigravity. The high-solubility sample can be directly diluted in the buffer that builds up a suitable environment to interact with the detection system, whereas a moderate- to low-solubility sample can be dissolved using a low-concentration organic solvent as a cosolvent. The sample slowly migrates to the conjugate pad from the sample pad. This contains target-specific antibody–reporter molecule conjugates. In cases where the conjugate pad was omitted, the antibody–reporter molecule conjugates were mixed into the sample with an appropriate amount [33, 34, 45–47].When the liquid passes through the conjugates reservoir, the antibody–reporter molecule conjugates are slowly released. Few antibodies start to bind with the antigen and move together to the test zone. Test zone is normally filled with hapten–carrier conjugates. Under a high analyte concentration, most antibody–reporter molecule conjugates are filled with analytes in the antibody binding sites. Thus, the antibody is unable to bind the hapten–carrier conjugates leaving no spot on the strip. Conversely, there is space on the binding sites of antigen–reporter molecule conjugates, which can react with the antigen of immobilized hapten–carrier molecules under a low analyte of interest condition. The accumulation of the reporter molecules on that zone renders the spot visible by color development or fluorescence (depending on the type of reporter molecules). Afterward, the liquid passes through the last zone called the “control zone.” This zone is used to indicate the suitability of the system. It is normally immobilized by the antibody, which can react to the antibody–reporter molecule conjugates whether the binding sites are free or not. Thus, in this zone, a spot is always present under the immunochromatography-appropriate condition. The shape, color, and intensity of this zone indicate the abnormalities of the strip test system. The test and control zones can be designed in a band or spot-shape. However, the band-shape zones require special equipment to evenly transfer the designated solution onto the desired area [48]. The in-house reagent dispensing machine can be used with proper validation [48–50]. Dissimilar to the spot shape zones, simple biotechnology instruments, such as the pipette, can be used where access to the lateral flow dispenser is limited. The size and shape of the zones can be designed based on the function between the area of the zone and the concentration of antibody–reporter molecule conjugates. The small area of the zones required a minimal number of antibody–reporter molecule conjugates leading to high sensitivity. However, an extremely small area of the zones leads to the invisibility of the zones. Thus, the zone design should be optimized in the individual test. The excess liquid of the system is adsorbed in an adsorbent pad. The adsorption protects the unwanted backflow of the liquid in the strip. Generally, the band or spot with the intensity difference can be determined via visual observation. The appropriate reader or interpretation software is used for the intensity measurement in cases where thoroughly precise results are needed.

#### Multiplex competitive format

**Fig. 3:**
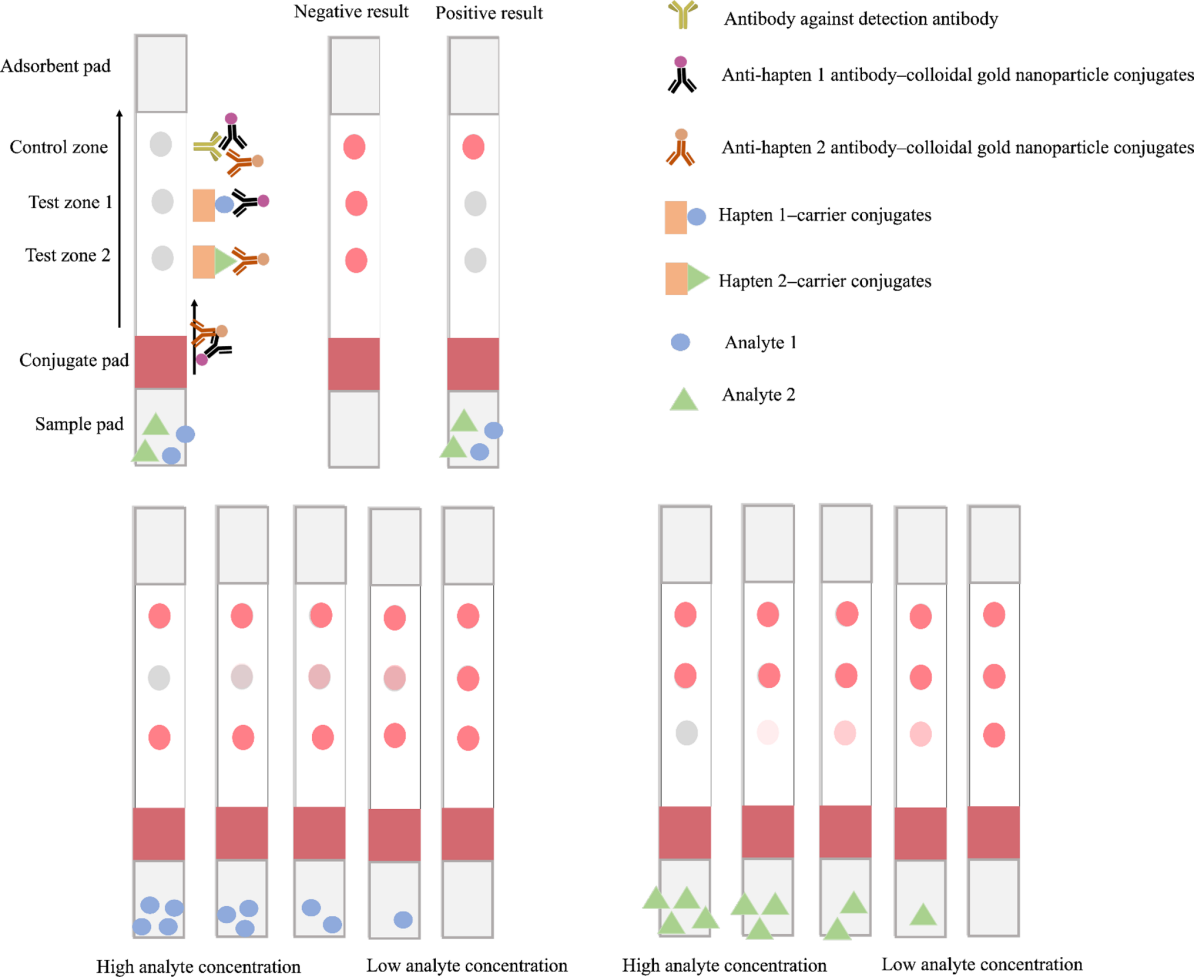
Multiplex competitive format of the strip test. The figure illustrates the multiplex competitive format designed for the simultaneous detection of two analytes. The upper right part demonstrates the symbolic representation of each compartment. The figure demonstrates the strip in various concentrations of analyte 1 and 2

More than one analyte can be detected on an individual strip, as shown in Fig. 3. The principle of this format is identical to the general competitive format. However, more than one antibody that corresponded to the number of analytes of interest are required in this system. The antibodies are individually embedded on the detection probe in the separated environment and applied in the same conjugate pads or test solution. In the general competitive format, there is only one test zone on the strip test, whereas the multiplex competitive format has more than one. The system incorporates the advantages from the multiple antibodies that render simultaneous detection possible in one analysis. The signal reporter elements can be the same (e.g., colloidal gold and colloidal gold) or different (e.g., colloidal gold and carbon nanoparticle) in one system. However, the selection of the nanoparticle for simultaneous detection presents many points of concern, particularly for compatibility among nanoparticles. The cross-reaction of the antibody is also a point of concern. The selected antibodies should not be bound to identical small compounds. This format can be extremely useful for controlling the quality of certain plants. The simultaneous detection system was first developed for the quality control of ginseng, in which the ginsenoside Rb1 and ginsenoside Rg1 (major biologically active compounds isolated from *Panax ginseng*) were used as analytes [51]. A sensitive detection system has been developed, regardless of the structural similarity between these analytes. According to the Japanese pharmacopoeia 18th edition (JP18th), the qualified *Panax ginseng* required the appropriate amount of ginsenoside Rb1 and ginsenoside Rg1 at 0.20% and 0.10% (w/w dry weight), respectively [52]. Therefore, the benefit of the strip test for screening the raw material is highlighted and fits the pharmacopoeia criteria. Nowadays, identical principles are used for several analytes, and this method is called microarray detection [53]. However, this concept has never been applied to phytoproducts.

#### Sandwich format

**Fig. 4:**
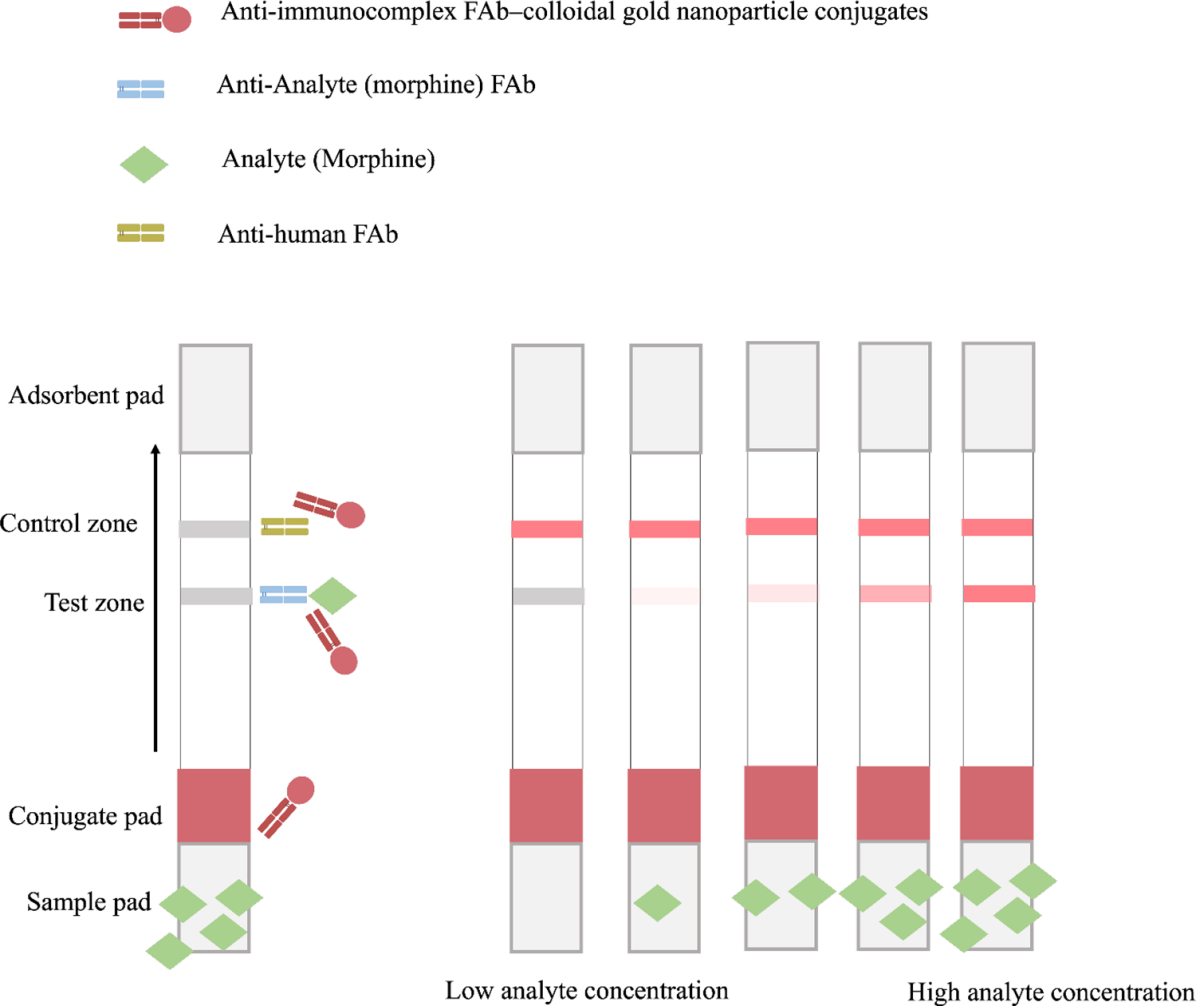
Sandwich format of the strip test. The upper section demonstrates the symbolic representation of each compartment. The figure demonstrates the LFA signal in various analyte concentrations. This figure was summarized from Teerinen et al. [58] where morphine was detected

Apart from the competitive basis of immunochromatography, there is a sandwich immunochromatography format for small-molecule detection. Superior specificity is expected when the sandwich system is applied since the result appeared when two different antibodies recognized two different epitopes on the same antigen. The sandwich format of the LFA has generally been applied for a relatively large molecule, such as a microorganism cell [54] and protein [55]. This is because the sandwich system requires two distinct antibodies, which can bind to different epitopes. However, obtaining the appropriate pair of antibodies for secondary metabolites or an adulterant containing small molecules consumes several attempts and resources. This may be due to the steric hindrance blockage between the antigen–capture antibody and antigen–tag antibody [56]. In extremely small molecules, this format is practically impossible to develop because the epitope position was shorter than 5 Å (the chain length of 5 carbon atoms), which was supposed to exhibit a minute chance of success [57]. However, a study has successfully performed the LFA using this format with modifications, as demonstrated in Fig. 4. The liquid flow of this format is identical to the competitive assay; however, the test zone was altered. The morphine-detectable LFA was developed by placing the anti-morphine fragmented antibody (FAb) on the test zone as a capture antibody [58]. When the morphine in the sample passed through the anti-morphine FAb, the morphine molecule was bound to the anti-morphine FAb generating the immunocomplex (FAb-morphine complex). The reporter molecule (gold nanoparticle) that conjugated with the anti-immunocomplex FAb was used as a detection antibody. When the immunoprobe passed through the immunocomplex on the membrane, the reporter molecules were accumulated, and the visible band was shown on the strip. The higher the morphine content in a sample, the higher the intensity of the visible band obtained, which was the opposite read-out style, compared with that of the competitive format. Although the system is specific, sensitive, and easy to interpret, the format is not quite famous as it has not been applied for another natural compound or plant product adulterant, as far as we know. This may be due to the complex steps of dual antibody preparation and the time-consuming preparation of two antibodies.

### Type of analysis in LFA

The LFA could be developed for qualitative, semi-quantitative, and quantitative analyses, up to the design of that individual study. Generally, LFA for phytoproducts is competitive format as mentioned in the previous section. Thus, type of analysis in LFA would discuss based on this format hereafter. Table 1 summarizes the detail of each type of analysis.

**Table 1:** LFA analysis type

	Analysis type		
	Qualitative	Semi-quantitative	Quantitative
Detection method	Visual observation	Visual observation with comparison with standard	Strip reader
		Photo analysis software (optional)	Scanner with photo analysis software
Results	Yes/no results	High-medium–low results	Certain amount/concentration results
Results interpretation	From presence/absence of the spot	From estimated intensity of the spot	From the intensity value which fits in the determination curve

#### Qualitative analysis

This detects the presence or absence of the compound in the matrix; however, it cannot describe the concentration level of the compound. A positive result, absence of spot, in this assay type indicates that the analyte concentration is more than the limit of detection (LOD) of the system, whereas a negative result, presence of spot, indicates that the analyte concentration is lower than the LOD or that the compound is not present in the sample. Although the benefits of this analysis type are limited, most assays are developed for qualitative analysis because the result interpretation is simple and exhibits low variation among interpreters. Considering that the presence of spot is the result indicator, there is no requirement for special equipment or software for the interpretation. Moreover, the qualitative analysis is suitable for compound-rich and color-rich matrixes, such as plant extracts. Occasionally, the interference of the matrix leads to a malfunction of the antibody. Hence, defining the accurate amount of analyte is difficult because the intensity of the spot fluctuates from matrix to matrix. Additionally, the vivid or dark color of the extract could interfere with the results, when accurate intensity is required. However, the presence or absence of the spot can be easily justified.

#### Semi-quantitative analysis

This analysis provides the estimation of the concentration of an analyte in the form of levels. The results are usually categorized as the level of concentration of the analyte in a sample into high (+++), medium (++), low (+), and very low/absence (–) considering that the intensity of the spot is subjective. Thus, the intensity comparator is required to reduce the bias of the interpretation. To elucidate the interpretation, certain studies that applied the competitive format designated the absence of a spot as high concentration, a weak intensity spot as low concentration, and a strong intensity spot as an extremely low concentration or absence of analytes [34].

#### Quantitative analysis

Only few studies developed the LFA as a quantitative analysis tool because it requires more validation processes and special equipment for interpretation. The results obtained from this analysis are more detailed, and the exact amount of the analyte can be reported. The method validations to obtain a reliable standard curve are required besides the qualitative analysis validation methods.

### Strengths and weaknesses of the LFA

The strengths and weaknesses of the LFA are described in Table 2. The use of LFA for phytoproduct analysis provides various benefits over other detection methods. The main dominant point of the LFA is that it can be readily performed at the point of need because it is extremely portable. Most prepared strips require only an apply-and-interpret step. This reduces the need for skillful labor. The LFA generally requires a shorter time for one analysis when compared with the ELISA or conventional chromatographic methods. For certain systems, the liquid sample can be directly applied onto the strip without any sample pretreatment. Considering that the assay is based on the antibody and antigen reaction, a highly specific antibody can be used to obtain a highly analyte-specific assay. The already produced strip can be preserved and used when required under various conditions, even at room temperature [59]. The shelf life of the prepared strip is generally longer than that of the ELISA kit, which is usually prepared in liquid form. The strip can be easily scaled-up for a large batch production. The cost of all materials for one assay is reasonable. The signal can be simply interpreted by visual observation. Moreover, a signal reader is not required for the qualitative system. Thus, the reach ability of the assay is high, particularly in developing countries. The system is designed for single use. Thus, the possibility of contamination by a previous assay sample, which occurs in the conventional column chromatographic methods, is diminished. Moreover, the need for equipment sanitization is minimized. Compared with the conventional column chromatographic methods, this assay requires an extremely small amount of organic solvent, indicating that it is more environmentally friendly and safe for the user.

**Table 2:** Strengths and weaknesses of the LFA for phytoproducts analysis

Strengths	Weaknesses
Ready to use	High cost and high labor intensive for antibody production
Rapid device preparation time	Non-specific binding of antibody possibly occurs
Simple analytical procedure	Questioning reproducibility (especially lot-to-lot)
Analyte-specific method	Yes/no results output
Shorter analytical time required compared to conventional chromatographic methods and ELISA	Comparator for semi-quantitative analysis is required
No sample pretreatment step needed	The sensitivity is generally lower than ELISA
Controlled storage condition is not required	
Easy to scale-up	
Cost-effective method	
Easy to convert the signal to value	
Applicable in many scenarios	
No signal reader needed	
No previous sample contamination	
Low organic solvent requirement	
Low sample volume needed	

Although the LFA has various strengths, there are limitations. The specific antibody is the fundamental requirement of the assay, and the cost and labor for antibody production are relatively high. The nonspecific binding of the antibody occurs because of the cross-reaction profile of an antibody or by skipping the washing step in the LFA. The detection protocol generally does not involve washing the strip. The strip test is generally designed for “positive/negative” results. For the semi-quantitative and quantitative analyses, the intensity of the spot is difficult to achieve a subjective judgment. This limitation can be overcome with the use of a color intensity analytical software with input from membrane strip readers, scanners, or digital cameras. If a semi-quantitative analysis is required, the strips applied with gradient concentrations of the analyte are required for comparison. Dissimilar to the ELISA, this system generally has no signal amplifier, such as horseradish peroxidase. The system requires several molecules of the antibody–nanoparticle conjugates to provide a sufficient signal for reading. This results in lower sensitivity when compared with that of the ELISA.

### Production of anti-hapten antibody

**Fig. 5:**
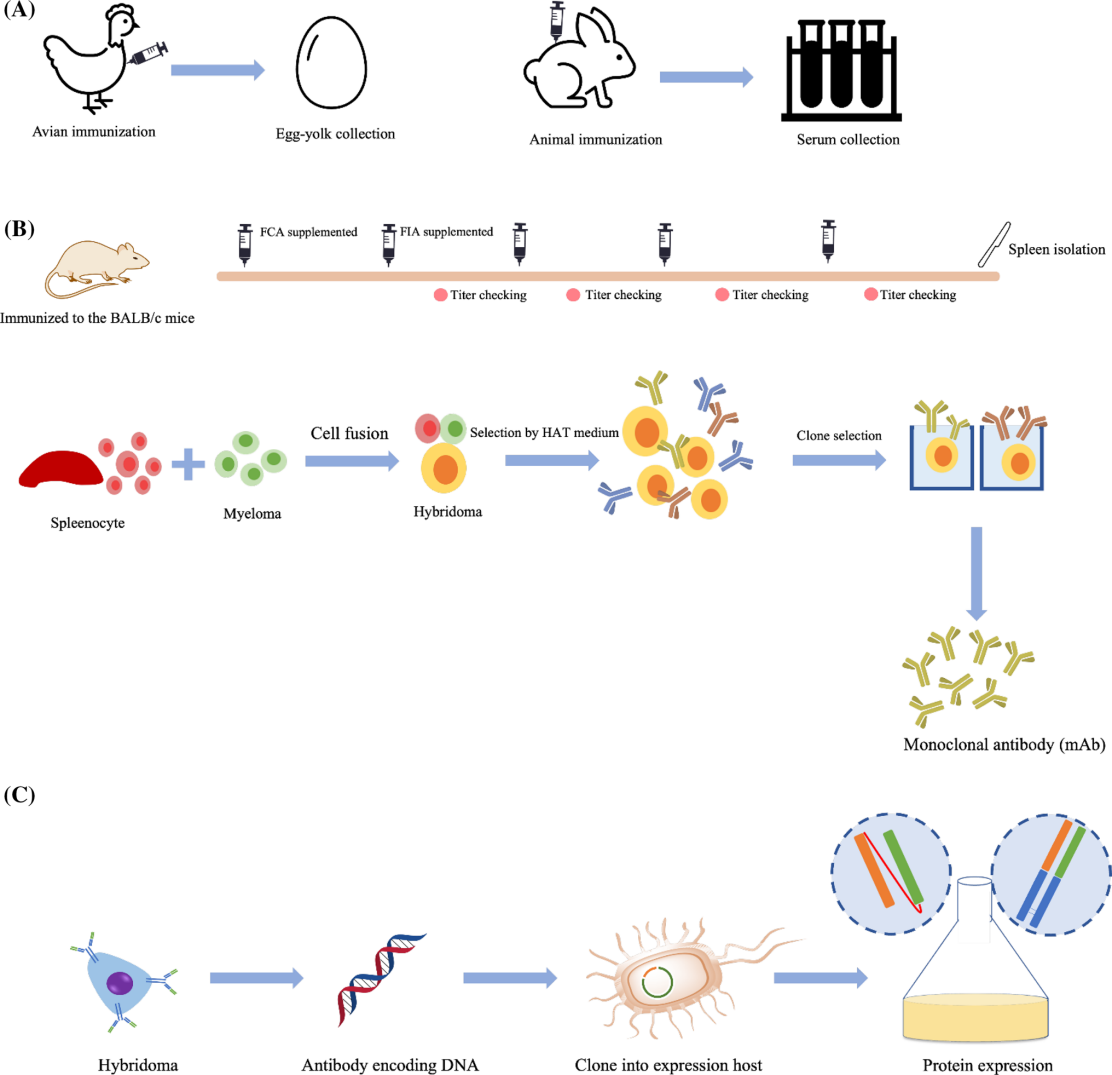
Summary of antibody production for the LFA applied for phytoproducts. **A** Production of pAb. In the avian host, polyclonal IgY is typically produced from the immunization of the avian; the egg yolk containing polyclonal IgY is collected and purified, and the serum containing polyclonal IgG is collected in the bigger animal, e.g., rabbit. **B** Production of mAb through the hybridoma technique. The animal host (mouse), as shown in the figure, was sequentially immunized, and the spleen was collected for cell fusion. The selection process using hypoxanthine–aminopterin–thymidine (HAT) medium and limited dilution enabled the desired characteristic hybridoma to be expanded. The mAb was produced from the supernatant of the selected clone. FCA and FIA represent Freund’s complete adjuvant and Freund’s incomplete adjuvant, respectively. **C** Production of recombinant antibody using bacteria as a host. The gene encoding the antibody was fragmented and cloned into the bacteria. With the appropriate expression technique, the fragmented antibody, i.e., FAb or single-chain variable fragment was produced

The unique structure glycoprotein called immunoglobulin (Ig) is the key component of the LFA as a detection tool. The antigen-specific antibody is secreted from the B-cell lymphocytes as a response of adaptive immune systems. The polyclonal antibody (pAb) and monoclonal antibody (mAb) are applied in LFA. However, mAb is the major type of antibody used for phytoproducts analysis LFA because its specificity and sensitivity are selectable, and the batch-to-batch quality of the antibody is controllable [60]. Regarding an avian-based antibody, the egg-derived polyclonal IgY antibody was applied in strip test fabrication [42]. The major isotype frequently applied in LFA is IgG because of its superior sensitivity, less cross-reactivity, and minimally complicated purification process, compared with another isotype. Figure 5 shows the summary of antibody production for LFA applied for phytoproducts.

The antibody production of macromolecules (Mw > 10,000 Da) is simple because the macromolecules frequently exhibit strong antigenicity. Plant secondary metabolites or contaminants are typically small-molecule compounds (Mw < 900 Da), which do not exhibit antigenicity. Therefore, various types of carriers have been used to enable these small molecules to exhibit antigenicity. Keyhole limpet hemocyanin (KLH) and BSA are the carriers of choice in the production of anti-small-molecule antibodies because these proteins possess many functional groups, which can be easily conjugated with small-molecule compounds using few steps [61, 62]. Moreover, these proteins are relatively large (KLH, 350 kDa; BSA, 66.5 kDa), compared with the small molecules. Thus, the immune system of the host animal can recognize, and the antibody can be successfully produced. The hybridoma formation is widely used when the production of mAb is required. There are three types of antibodies, which are predicted to be produced by the hybridoma, including the anti-carrier, anti-hapten–carrier complex, and anti-hapten antibodies. To sieve out the unwanted antibodies (anti-carrier and anti-hapten–carrier complex), the structurally different carrier conjugates, such as the human serum albumin (HSA), ovalbumin (OVA), mouse serum globulin, thyroglobulin, and diphtheria toxoid can be applied for antibody screening. The structurally different carrier conjugates can be used to develop the immunoassay when the antibody recognizes certain parts of the immunized molecules.

Recombinant antibody fragments are a group of antibodies that are not widely applied in the LFA because of their limited sensitivity and stability, compared with its parent antibody [63]. Moreover, the probability that the binding site of the antibody could bind to the detection probe was high because the molecules of these fragment antibodies were smaller. However, the ease of antibody production by *Escherichia coli* makes the recombinant antibody format attractive. Recently, a recombinant FAb was used as a single detection antibody to detect deoxymiroestrol, the potent phytoestrogen isolated from *Pueraria candollei*, in plant samples [64]. Noteworthily, the specificity and sensitivity profiles of this produced FAb were different from that of the original mAb [65]. The FAb tended to be more specific to one antigen rather than cross-reactive to structurally related compounds in this particular study. Therefore, the sensitive LFA of deoxymiroestrol was developed using these advantages.

### Reporter for LFA

The labeling or reporter particle is the component that indicates the effectiveness and efficiency of the LFA. The antibody-directed particle accumulation generates a detectable signal on the membrane. There are various types of reporter particles applied in LFAs, such as colloidal gold nanoparticles, latex beads [66], carbon nanoparticles [67–69], composite nanoparticles [70], magnetic nanoparticles [71], liposomes [72], fluorescent probes [30, 42], and enzymes [73]. However, LFA for phytoproducts shared a common reporter molecule (colloidal gold nanoparticles), whereas a few used quantum dots or carbon nanoparticles as reporters. Table 3 lists the nanoparticles usually applied in LFAs for phytoproducts.

**Table 3:** Nanoparticles used in LFA development applied for phytoproducts

Nanoparticles	Color	Detection method	Strength	Weakness
Colloidal gold nanoparticles	Red-pink	Visual observation	Ease of labeling (physical adsorption)	Less color intensity lead to low sensitivity
		Strip reader (quantitative analysis)	Manageable surface	False positive and false negative possibly occur
		Photo analysis software (optional)	Widely used	Performance in red-yellow colored samples is low
			Commercially available	
			Ease of in-house synthesis and functionalization	
			Stable in various LFA condition	
Quantum dots	Depend on the material	Fluorescence strip reader	Ease of labeling (chemical conjugation)	Expensive
		Visual observation (optional/lower sensitivity)	Low photo-bleaching	Fluorescent strip reader needed
			Stable in various LFA condition	Toxic to the environment
			High sensitivity	
			Wide range absorption spectra	
			Specific emission wavelength	
			Photostability	
Colloidal carbon	Black	Visual observation	Ease of labeling (physical adsorption)	Performance in black colored samples is low
		Photo analysis software (optional)	Ease of In-house synthesis	
			Cost-effective	
			Stable in various LFA condition	
			Low toxicity	
			High contrast results (black and white)	

#### Colloidal gold nanoparticles

**Fig. 6:**
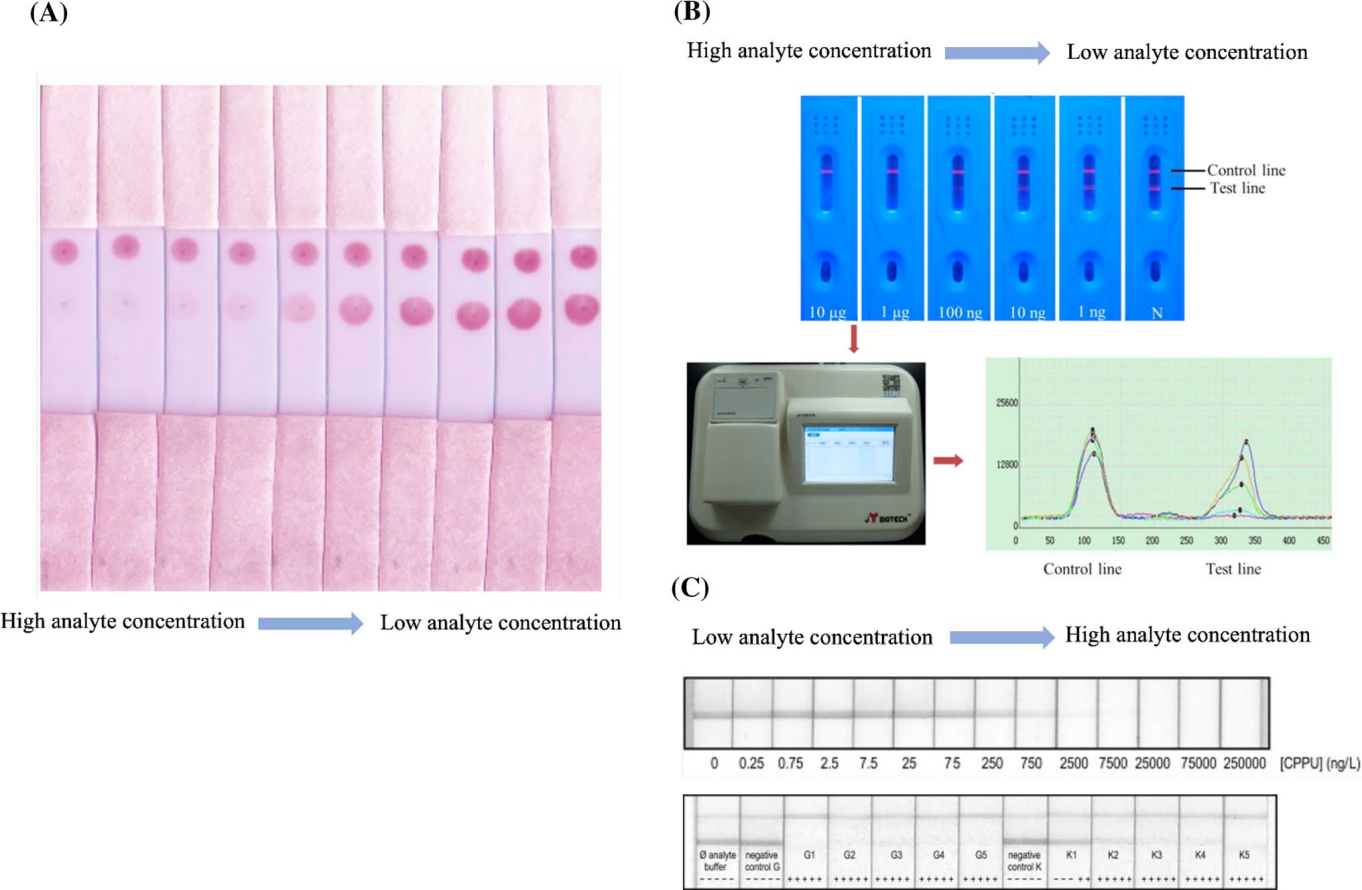
Signals obtained from different nanoparticles. **A** Signal obtained from the colloidal gold nanoparticles. Visual observation was applied for analysis. The picture was adopted from Nuntawong et al. [34]. **B** The signal obtained from the quantum dots. The upper section shows the signal of various analyte concentrations. The lower section shows the signal-reading machine and response curve of the signal. The picture was adopted from Qu et al. [37]. **C** Signal obtained from the carbon nanoparticle. The upper section shows the signal of various analyte concentrations. The lower section is the real sample application of the lateral flow immunoassay. The picture was adopted from Suárez-Pantaleón et al. [88]

This is the fluid or suspension form of the gold usually suspended in a water-based solution. The scarlet suspension is typically preferred over the blue or purple suspension as the particle size is less than 100 nm. The particle size of the nanoparticles can be selected according to the preference of the user. However, the oversize particles lead to the aggregation and sedimentation of the colloidal gold nanoparticles, whereas extremely small particles cause difficulty in the particle washing step and produce a meaningless color on the detection zones. The colloidal gold with a particle size exceeding 20 nm generated an interpretable signal [74]. Nevertheless, the appropriate particle size was proposed as approximately 40 nm because the maximum color was obtained at this size with the less steric hindrance of the antibody conjugation [74]. Colloidal gold is generally the first choice of material, owing to its various benefits. The labeling of the antibody to the colloidal gold can be performed by simple incubation. The antibody can bind to the colloidal gold particle through passive adsorption where the electrostatic force, hydrogen bonding, hydrophobicity, and Van der Waals forces are the main interactions [75, 76]. Moreover, the direction of the antibody (position of constant and variable regions) on the colloidal gold nanoparticles is manageable with surface modification [77]. Compared with other materials, the colloidal gold nanoparticles are relatively inexpensive. The particles are stable in solution or dried form. Additionally, the important characteristic of the colloidal gold nanoparticles is their long-lasting color on the membranes. The colloidal gold can be easily synthesized using various methods, and the surface functional group can be synthesized by preference [78, 79] and is easy to obtain through various commercial sources. When labeled with this nanoparticle, the assay results can be read-out on the basis of the colorimetric method. Nevertheless, the weaker signal intensity, compared with that of other nanoparticles is a point of concern. Generally, for the competitive immunoassay, the antibody concentration is important for the entire assay. The less the concentration of the antibody used in the system, the more sensitivity the developed system can be achieved. However, the colloidal gold nanoparticle–antibody conjugates concentration should be increased, when the signal is too weak to be interpreted. Moreover, false positives or negatives can occur in certain situations, such as environments with excess salt or extreme pH. The given color on the zones of the strip is pinkish red. It is not suitable to apply these nanoparticles in a test, where the sample is colored in the same tone, such as anthocyanin-, carotenoid-, and flavonoid-rich samples. Figure 6A demonstrates an example of a signal obtained from these nanoparticles.

#### Quantum dots

These are semiconductor nanoparticles; their optoelectronic properties are dependent on their composition. Generally, the particle size of quantum dots is in a range of 1.5–10 nm [80]. They exhibit high colloidal stability. Moreover, the material has a low rate of photobleaching and high chemical stability. A few studies have used quantum dots to develop the LFA for phytoproducts [37, 42, 81]. This may be due to the high price of the materials. However, the high sensitivity assay was obtained as a trade-off for this drawback. Although the quantum dot-based LFA can provide a colorimetric read-out on the test zone, the visual observation is not usually used for final result analyses because of the sharp drop-off in the sensitivity. The fluorometric approach is typically used for such assay detections. Therefore, the user needs a specific strip reader to read the results. The antibody labeling can be easily conducted using simple carbodiimide-mediated methods or commercially recommended methods, depending on the functional group at the surface of the quantum dots and element of the antibody (Fv and Fc) preferred for conjugation. Compared with the organic dye labeling e.g., fluorescein and R-phycoerythrin, quantum dots exhibit superior photostability, high fluorescence, and broad adsorption spectra with specific emission wavelength [82]. Thus, quantum dots are useful as a good candidate for LFA development. They were first used to develop the LFA for the detection of puerarin (kakonein), the biologically active isoflavone isolated from the root of *Pueraria lobata* [37]. The system exhibited impressive sensitivity as the detection limit was 5.8 ng/mL, which was above the average sensitivity of other LFAs developed for phytoproducts until 2016. The production process of quantum dots and the material itself are usually toxic to the environment. Hence, proper waste management measures should be in place. Figure 6B demonstrates an example of a signal obtained from these nanoparticles.

#### Carbon nanoparticles

These can be called colloidal carbon or carbon black. As they are intense-colored particles, the colorimetric method is usually used for read-out. The particles are easy to prepare for in-house use and are provided in various commercially available forms [83, 84]. There are a few forms applied in LFAs, such as nanostrings [67, 85] and nanotubes [86]. The carbon nanoparticles are extremely stable in various chromatographic environments. Moreover, their toxicity is relatively lower than that of quantum dots. The conjugation of the antibody to the probe is performed by simple incubation without any modification (physical adsorption) [67]. The results can be evaluated by visual observation because the black spot provides high contrast to the white color of the membrane [87]. These nanoparticles have been used in many fields for a decade. However, their application in plant sample detection is limited. Forchlorfenuron, the synthetic cytokinin usually spiked in agricultural products for regulating plant growth, was determined using carbon nanoparticles as an immunoprobe [88]. This LFA was developed to quantify the concentration of an analyte, which corresponded to the gray color measured using photo analysis software. The limit of quantification was 89 ng/L in an optimized buffer and 33.4 mg/kg in a kiwi and grape matrix. Although the carbon nanoparticles possess versatile benefits, black compounds and black matrixes should not be used on the strip developed by these nanoparticles. Figure 6C demonstrates an example of a signal obtained from these nanoparticles.

### Production of hapten–carrier molecules

Plant-derived compounds and contaminants are mostly small compounds. Small compounds are difficult to be immobilized to solid phase (well-plate and membrane) without pretreatment. Thus, hapten–carrier protein conjugates are generally used as an immobilizable antigen. There is no carrier of choice in LFAs for phytoproducts. The best carrier protein is selected on the basis of the experiment. The shape, size, and intensity of the band or spot in LFA per concentration unit are important factors. Small molecules can be conjugated with carriers with or without linkers. There are no solid rules in hapten–carrier design. Thus, an appropriate conjugation method for an individual hapten should be developed. Small molecules were routinely conjugated with carriers by simple chemical reactions (carbodiimide-mediated, sodium periodate, and Mannich reactions), as summarized in Table 4. The selection of these reactions depends on the structure of the target molecule. Considering that the reactive aldehyde is easily formed at the vicinal diol of the sugar part, which readily reacts with the amine group of the protein carrier, the sodium periodate reaction is typically preferred in the conjugation of sugar-containing compounds [37, 39, 42, 51, 89–94]. However, the cross-reaction of the antibody against the aglycone of the glycosides should be considered when the antigen is prepared by this reaction. The compounds with carboxylic acid and hydroxyl functional groups are usually conjugated by a carbodiimide-mediated reaction [33, 34, 47, 95–102], whereas compounds with active hydrogen, e.g., α-picolines, ketones, esters, and acetylenes, can be conjugated to an amine or amide group of the carrier by a Mannich reaction [103, 104]. Furthermore, structure modifications of the parent compounds to add the reactive functional group to the molecule can be conducted when necessary.

**Table 4:** Conjugation methods of the hapten–carrier protein used in LFA for phytoproducts

Coupling method	Reagent	Analyte	Carrier	References
Carbodiimide mediated method	CDI	Monocrotaline	HSA	[33]
	CDI	Kwakhurin	BSA	[102]
	CDI	(*S*)-Higenamine	Gamma globulin	[34]
	CDI	Amarogentin	HSA	[46]
	DCC	Glycyrrhizin	BSA	[97]
	EDC	Sennosides A and B	HSA	[96]
	EDC	Baicalin	BSA	[98]
	EDC	Mitragynine	OVA	[99]
	EDC	Salvinorin A	HSA	[100]
	EDC	Mitragynine and 7-hydroxymitragynine	BSA	[101]
	EDC	Aristolochic acid I	BSA	[47]
Mannich reaction	Formaldehyde	Miroestrol	OVA	[103]
	Formaldehyde	Isomiroestrol	Cationized OVA	[104]
Sodium periodate	NaIO_4_	Ginsenosides Rb1 and Rg1	HSA	[51]
	NaIO_4_	Glycyrrhizin	HSA	[89]
	NaIO_4_	Pseudojujubogenin glycosides	HSA	[90]
	NaIO_4_	Asiaticoside	HSA	[91]
	NaIO_4_	Mulberroside A	OVA	[92]
	NaIO_4_	Puerarin	BSA	[37]
	NaIO_4_	Daidzin and genistin	HSA	[93]
	NaIO_4_	Harringtonine	BSA	[45]
	NaIO_4_	Miroestrol and puerarin	HSA	[94]
	NaIO_4_	Saikosaponin d	BSA	[39]
	NaIO_4_	Rhein	OVA	[42]
	NaIO_4_	Colchicine	OVA	[44]

*CDI* carbonyldiimidazole, *DCC* *N,N*'-dicyclohexylcarbodiimide, *EDC* 1-ethyl-3-(3-dimethylaminopropyl)carbodiimide, *HSA* human serum albumin, *BSA* bovine serum albumin, *OVA* albumin from egg white

### LFA developed to detect secondary metabolites in plant samples

There are various important components in plants, such as proteins, polysaccharides, and small-molecule secondary metabolites. However, small-molecule secondary metabolites have gained attention because they were observed to be unique in particular plant species and had a high potential to be biologically active compounds. LFAs can be used on virtually all small-molecule secondary metabolites, such as triterpenoid glycoside, benzylisoquinoline alkaloid, and flavone glycoside, as listed in Table 5. The main purpose of developing the assay is to inspect the quality of plants by investigating the amount of biologically active and toxic compounds in the plants. The concept of small-molecule detection by LFA was first introduced in 1996 when the analyte was progesterone (Mw = 314.46) [105]. The test zone was filled with an antiprogesterone antibody, and the detection probe was gold-labeled progesterone–OVA conjugates [105]. The strip functioned on a competitive basis, as mentioned in the “general principle of the LFA” section; however, the position of the main components (antibody and hapten–carrier protein conjugates) was switched. On the basis of this concept, the first LFA for ginsenosides Rb1 and Rg1 was invented [51]. Interestingly, no significant matrix effect that can alter the results was observed in this study, although the plant sample was extracted with an organic solvent and simply diluted before use. This advantage was observed in other studies and might be due to the affinity of the antibody to the antigen. Generally, the antibody can detect target compounds within the ng/mL to µg/mL level when it is applied to LFA. Therefore, it is not necessary to use a high-concentration sample. This ensures that the sample impurities that might interfere with the detection result (if any) are at a minimal level by simple dilution. Table 6 summarizes the plant secondary metabolite detection by the LFA.

**Table 5:** Plant secondary metabolites which applied in LFA

Secondary metabolites	Compounds classification	Plant resource	References
Ginsenosides	Triterpenoid glycoside	*Panax* spp.	[51, 106]
Sennosides A and B	Hydroxyanthracene glycoside	*Rhem* spp., *Senna* spp.	[96]
Glycyrrhizin	Triterpenoid glycoside	*Glycyrrhiza* spp.	[89, 97]
Pseudojujubogenin glycosides	Pseudojujubogenin glycoside	*Bacopa monnieri*	[90]
Asiaticoside	Triterpenoid glycoside	*Centella asiatica*	[91]
Baicalin	Flavone glycoside	*Scutellaria baicalensis*	[98]
Morphine	Alkaloid	*Papaver somuniferum*	[58]
Mulberroside A	Stilbene glucoside	*Morus alba*	[92]
Puerarin	Isoflavone glycoside	*Pueraria lobata*	[37]
Daidzin and genistin	Isoflavone glycoside	*Glycine max*	[93]
Miroestrol	Chromene	*Pueraria mirifica*	[103]
Harringtonine	Alkaloid	*Cephalotaxus harringtonia*	[45]
Monocrotaline	Pyrrolizidine alkaloid	*Crotalaria* spp.	[33]
Mitragynine	Indole alkaloid	*Mitragyna speciosa*	[99, 101]
Salvinorin A	Diterpenoid	*Salvia divinorum*	[100]
Saikosaponin d	Triterpenoid glycoside	*Bupleurum falcatum*	[39]
Icariin	Flavone glycoside	*Epimedium *spp.	[40]
Isomiroestrol	Chromene	*Pueraria mirifica*	[104]
Deoxymiroestrol	Chromene	*Pueraria mirifica*	[64]
Triptolide	Diterpenoid epoxide	*Tripterygium wilfordii*	[38]
Aristolochic acid I	Nitrophenanthrene	*Aristolochia* spp.	[47]
Aconitine	Alkaloid	*Aconitum* spp.	[29]
Rhein	Anthraquinone	*Rheum officinale*	[42]
Kwakhurin	Isoflavonoids	*Pueraria mirifica*	[102]
Higenamine	Benzyltetrahydroisoquinoline alkaloid	*Aconitum carmichaelii, Asarum heterotrophies, Nandina domestica*	[34]
Amarogentin	Secoiiridoid glycoside	*Swertia* spp., *Gentiana* spp.	[46]
Colchicine	Alkaloid	*Colchicum autumnale*	[44]

**Table 6:** Summary of the LFA used for plant secondary metabolites analysis

Analyte	Format	Antibody	Reporter molecule	Reading method	Assay type	Detection range/detection limit	Confirmation method	References
Ginsenosides Rb1 and Rg1	Multiplex competitive	mAb	Colloidal gold nanoparticles	Visual observation	Qualitative analysis	2 μg/mL for both analytes	icELISA	[51]
Sennosides A and B	Multiplex competitive	mAb	Colloidal gold nanoparticles	Visual observation	Qualitative analysis	125 ng/mL for both analytes	icELISA	[96]
Glycyrrhizin	Competitive	mAb	Colloidal gold nanoparticles	Visual observation	Qualitative analysis	250 ng/mL	icELISA	[89]
Glycyrrhizin	Competitive	mAb	Colloidal gold nanoparticles	Visual observation	Qualitative analysis	20–50 ng/ml	icELISA	[97]
Pseudojujubogenin glycosides	Competitive	mAb	Colloidal gold nanoparticles	Visual observation	Qualitative analysis	125 ng/mL	icELISA	[90]
Asiaticoside	Competitive	mAb	Colloidal gold nanoparticles	Visual observation	Qualitative analysis	12.5 μg/ml	icELISA	[91]
Baicalin	Competitive	mAb	Colloidal gold nanoparticles	Visual observation	Qualitative analysis	0.6 μg/mL	icELISA	[98]
Ginsenoside Re	Competitive	mAb	Colloidal gold nanoparticles	Visual observation	Qualitative analysis	200 µg/L	icELISA	[106]
Morphine	Sandwich	FAb	Colloidal gold nanoparticles	POCTER reader	Quantitative analysis	1 ng/mL	Not mentioned	[58]
Mulberroside A	Competitive	pAb	Colloidal gold nanoparticles	Visual observation	Qualitative analysis	2 µg/mL	icELISA	[92]
Puerarin	Competitive	mAb	Quantum dots	Strip reader	Qualitative and quantitative analysis	1–10 μg/mL; 5.8 ng/mL	HPLC–UV	[37]
Daidzin and genistin	Competitive	mAb	Colloidal gold nanoparticles	Visual observation	Semi-quantitative analysis	125 ng/mL	HPLC–UV	[93]
Miroestrol	Competitive	mAb	Colloidal gold nanoparticles	Visual observation	Qualitative analysis	0.156 µg	icELISA	[103]
Harringtonine	Competitive	mAb	Colloidal gold nanoparticles	Visual observation	Semi-quantitative analysis	39.1–313 ng/mL; 313 ng/mL	icELISA	[45]
Monocrotaline	Competitive	mAb	Colloidal gold nanoparticles	Visual observation	Qualitative analysis	0.61 ng/mL	icELISA	[33]
Mitragynine	Competitive	mAb	Colloidal gold nanoparticles	Visual observation; Photo analysis software	Qualitative analysis	1 mg/mL of mitragynine by visual assessment and 0.60 mg/mL by Image J analysis	icELISA, HPLC–UV	[99]
Salvinorin A	Competitive	mAb	Colloidal gold nanoparticles	Visual observation	Qualitative analysis	0.625 µg/mL	icELISA	[100]
Miroestrol and puerarin	Multiplex competitive	pAb (puerarin); mAb (miroestrol)	Colloidal gold nanoparticles	Visual observation	Qualitative analysis	Detection limits of miroestrol and puerarin are 0.15 μg and 4.5 μg, respectively	icELISA	[94]
Saikosaponin d	Competitive	mAb	Colloidal gold nanoparticles	Strip reader	Quantitative analysis	96 ng/mL–150 µg/mL	icELISA	[39]
Mitragynine and 7-hydroxymitragynine	Competitive	mAb	Colloidal gold nanoparticles	Strip reader	Qualitative and quantitative analysis	0.16–5 μg/mL	icELISA	[101]
Icariin	Competitive	mAb	Colloidal gold nanoparticles	Visual observation	Qualitative and quantitative analysis	500 ng/ml	icELISA	[40]
Isomiroestrol	Competitive	mAb	Colloidal gold nanoparticles	Visual observation	Qualitative analysis	7.0 µg/mL	icELISA	[104]
Triptolide	Competitive	mAb	Colloidal gold nanoparticles	Visual observation	Qualitative analysis	1 μg/mL	LC–MS/MS	[38]
Aristolochic acid I	Competitive	mAb	Colloidal gold nanoparticles	Visual observation	Qualitative analysis	0.25 μg/g	Not mentioned	[47]
Aconitine	Competitive	mAb	Colloidal gold nanoparticles	Visual observation	Qualitative analysis	10–25 ng/mL	Not mentioned	[29]
Rhein	Competitive	pAb IgY antibody	Quantum dots	Fluorescence-strip reader	Quantitative analysis	80–5000 ng/mL; 98.2 ng/mL	HPLC–UV	[42]
Kwakhurin	Competitive	mAb	Colloidal gold nanoparticles	Visual observation	Qualitative analysis	160 ng/mL	HPLC–UV	[102]
(*S*)-Higenamine	Competitive	mAb	Colloidal gold nanoparticles	Visual observation; Photo analysis software	Semi-quantitative analysis	156 ng/mL	icELISA, HPLC–UV	[34]
Amarogentin	Competitive	mAb	Colloidal gold nanoparticles	Visual observation	Semi-quantitative analysis	31.25–500 ng/mL; 250 ng/mL	icELISA	[46]
Colchicine	Competitive	mAb	Colloidal gold nanoparticles	Visual observation; Handheld strip scanning reader	Quantitative analysis	1–25 ng/mL in milk, 2.5–50 ng/mL in beef, 1–25 ng/mL in edible lily, and 2.5–25 ng/mL in daylily	HPLC-TOF	[44]
Deoxymiroestrol	Competitive	FAb	Colloidal gold nanoparticles	Visual observation; photo analysis software	Qualitative analysis	250 ng/mL	icELISA	[64]

#### Selectivity of the system

The selectivity of the LFA is an important factor that validates LFA results. The benefit of an antibody-based assay over the chromatographic-based assay is the selectivity. The molecular-level structure recognition of the antibody–antigen reaction is theoretically more selective than the conventional detectors, such as UV–Vis or fluorescent detectors. However, the cross-reactivity of the antibody against small molecules should be investigated. Considering that the matrix of the sample is usually the organic solvent extract of the plant of interest, there is a high chance that structurally similar compounds originating from the mutual biosynthesis pathway are present in the matrix. Furthermore, the glucoside form (aglycone target) or aglycone form (glycoside target) of the target compounds should be considered. The antibody that exhibits the cross-reaction might be useful when the cross-reacted compound is not present in the plant [34]. In certain cases, the broad cross-reaction of the antibody is useful when the class detection or total amount of the analyte and its cross-reacted compounds is desired [93]. Most antibodies applied in the LFA are well-characterized using icELISA before use. However, the cross-reactivity of the LFA system has been revised to ensure selectivity after conjugation with reporter molecules. Interestingly, the cross-reaction profiles obtained from LFAs correlate with the icELISA result in most LFAs applied for plant samples. This indicates that the cross-reaction profile of the antibodies is usually not altered by nanoparticle conjugation at least in LFAs applied for plant samples. The cross-reaction test is simply performed by challenging the developed LFA with cross-reaction compound candidates at a certain concentration. In certain cases, the percentage of cross-reaction (%CR) can be reported using the relative proportion of the LOD of the target compound and that of the candidates [33].

Additionally, the selection of the antibody type is important. Both pAb and mAb are applied in LFA. Similar to other antibody-based assays, pAb, which normally recognizes multiple epitopes, theoretically exhibit lower selectivity compared with mAb, which recognizes a single epitope and desired characteristics are precisely refined [13]. Hence, the antibody of choice for LFA is the mAb. Nevertheless, there are exceptions in certain haptens. For puerarin, pAb [94], and mAb [37] were applied in LFA and exhibited similar cross-reactivity profiles.

#### Sensitivity of the system

For LFA, the sensitivity indicates the usefulness of the system. Generally, the strip test is suitable for screening purposes. Hence, the sensitivity does not need to be as high as the confirmation analysis. However, optimization to obtain the highest sensitivity is recommended. The sensitivity of the LFA applied for plant samples is usually described as the LOD of the developed system. As mentioned in Table 7, the LOD of the LFA is usually higher than that of the ELISA-based method. Nevertheless, the sensitivity remains comparable or superior to the conventional chromatographic method in a few cases.

**Table 7:** Sensitivity comparison between LFA and icELISA

Analyte	LFA determination range/LOD	icELISA determination range/LOD
Ginsenosides Rb1 and Rg1	2 μg/mL for both analytes	Ginsenoside Rb1 and Rg1 are 20–400 ng/mL [107] and 0.3–10 µg/mL [108], respectively
Sennosides A and B	125 ng/mL for both analytes	Sennoside A and sennoside B are 20–200 ng/mL [109] and 0.5–15 ng/mL [110], respectively
Glycyrrhizin	250 ng/mL	20–200 ng/mL [111]
Glycyrrhizin	20–50 ng/ml	0.2–5.1 ng/mL [97]
Pseudojujubogenin glycosides	125 ng/mL	1.95–62.5 ng/mL; 0.5 ng/mL [112]
Asiaticoside	12.5 μg/mL	0.78–50 µg/mL; 6.2 µg/mL [113]
Baicalin	0.6 μg/mL	200 ng/mL–2 µg/mL; 100 ng/mL [114]
Ginsenoside Re	200 µg/L	0.08–0.7 µg/mL [114]
Morphine	1 ng/mL	N/A
Mulberroside A	2 µg/mL	0.17–15.62 μg/mL [115]
Puerarin	1–10 μg/mL; 5.8 ng/mL	10 ng/mL–1 μg/mL; 181.3 ng/mL [37]
Daidzin and genistin	125 ng/mL	1.95–62.5 ng/mL; 1.95 ng/mL [116]
Miroestrol	0.156 µg	10–780 ng/mL; 3.5 ng/mL [117]
Harringtonine	39.1–313 ng/mL; 313 ng/mL	0.76–48.8 ng/mL; 0.76 ng/mL [118]
Monocrotaline	0.61 ng/mL	48.8 pg/mL–3.13 ng/mL [119]
Mitragynine	1 mg/mL of mitragynine by visual assessment and 0.60 mg/mL by Image J analysis	32.92–250 μg/mL; 32.47 μg/mL [120]
Salvinorin A	0.625 µg/mL	0.0195–0.625 μg/mL; 0.0195 μg/mL [121]
Miroestrol and puerarin	Miroestrol and puerarin are 0.15 μg and 4.5 μg, respectively	Miroestrol and puerarin are 10–780 ng/mL; 3.5 ng/mL [117] and 0.02–12.5 μg/mL; 0.02 μg/mL [122], respectively
Saikosaponin d	96 ng/mL–150 µg/mL	156.25 to 5000.00 ng/mL; 148.41 ng/mL [123]
Mitragynine and 7-hydroxymitragynine	0.16–5 μg/mL	0.047–6 μg/mL [101]
Icariin	500 ng/mL	5–3125 ng/mL; 8.41 ng/mL [40]
Isomiroestrol	7.0 µg/mL	390–12,500 ng/mL; 323 ng/mL [124]
Triptolide	1 μg/mL	N/A
Aristolochic acid I	0.25 μg/g	IC_50_ = 5.02 ng/mL [47]
Aconitine	10–25 ng/mL	1.13–11.76 ng/mL [29]
Rhein	80–5000 ng/mL; 98.2 ng/mL	5–3125 ng/mL; 8.41 ng/mL [42]
Kwakhurin	160 ng/mL	1.53–48.8 ng/mL; 1.13 ng/mL [125]
(*S*)-Higenamine	156 ng/mL	7.81–125 ng/mL; 4.41 ng/mL [126]
Amarogentin	31.25–500 ng/mL; 250 ng/mL	1.95–62.5 ng/mL; 1.28 ng/mL [95]
Colchicine	1–25 ng/mL in milk, 2.5–50 ng/mL in beef, 1–25 ng/mL in edible lily, and 2.5–25 ng/mL in daylily	0.09–2.16 ng/mL [44]
Deoxymiroestrol	250 ng/mL	31.25–1,000 ng/mL; 30.80 ng/mL [127]

#### LFA developed to detect the chemical contaminants

The presence of unnatural contaminants in plants is a point of concern for consumers. There are various compounds spiked into crops for agricultural purposes. Herbicides and insecticides are used in the large-scale production of plants to control weeds and pests, thereby preserving the yield. Occasionally, the use of fungicides is necessary to combat fungal microorganisms to preserve the yield, shelf life, and quality of plants [12]. Phytoregulators are necessary for controlling the growth rate and growth stages (flowering and fruiting) of crops. Although these chemicals are necessary for agricultural purposes, the high intake of these chemicals can be harmful to consumers. Thus, a limited level of these compounds is set to ensure safe consumption. Several studies have attempted to promptly detect chemical contaminants in phytoproducts, as summarized in Table 8. Samples containing contaminants can be extracted by simple organic solvent extraction. The detection method is based on the competitive format. Noteworthily, the major developed strip tests for chemical contaminants are highly sensitive (several ng/mL for LOD) to meet the limit of contaminants in plant samples.

**Table 8:** Summary of the LFA used for phytoproduct contaminants analysis

Analyte	Classification	Format	Reporter molecule	Reading method	Detection range/LOD	Confirmation method	References
Tebuconazole	Fungicide	Competitive	Quantum dots	Visual observation; Fluorescent strip reader	0.02–1.25 ng/mL	LC/MS–MS	[81]
Prometryn	Herbicide	Competitive	Colloidal gold nanoparticles	Visual observation	1 ng/mL	icELISA; LC/MS–MS	[128]
Forchlorfenuron	Phytoregulator	Competitive	Carbon nanoparticles	Photo analysis software	89 ng/L in buffer, 33.4 mg/kg in kiwi and grapes	icELISA; HPLC–UV	[88]
6-Benzylaminopurine	Phytoregulator	Competitive	Colloidal gold nanoparticles	Visual observation	10 ng/g	Not mentioned	[129]
Imidaclothiz	Insecticide	Competitive	Colloidal gold nanoparticles combined with fluorescent-peptide tracer	Visual observation; Photo analysis software	8.00 ng/mL	HPLC–UV	[130]
Carbofuran	Insecticide	Competitive	Colloidal gold nanoparticles	Membrane strip reader	1 ng/mL	icELISA	[131]
Carbofuran, isoprocarb, carbaryl	Insecticide	Competitive	Colloidal gold nanoparticles	Membrane strip reader	carbofuran, isocarb, and carbaryl limit of quantification is 0.05 ng/mL, 31.3 ng/mL, and 31.3 ng/mL, respectively	Not mentioned	[132]
Parathion and triazophos	Insecticide	Competitive	Colloidal gold nanoparticles	Visual observation	The detection limits for parathion and triazophos were 0.1 µg/mL and 0.05 µg/mL, respectively	GC	[133]
Atrazine	Herbicide	Competitive	Colloidal gold nanoparticles	Visual observation	12.5 ng/mL (*Salviae miltiorrhizae* radix et rhizome), 12.5 ng/mL (Astragali radix), and 6.25 ng/mL (Isatidis radix)	dcELISA; LC–MS/MS	[134]

## Conclusion

Phytoproducts are important for various industries. The small molecules in these products were highlighted because they are a quality indicator for phytoproducts. Reliable methods for controlling plant quality through qualitative and quantitative analyses of plant secondary metabolites and contaminants have been developed. Herein, LFA was discussed in the perspective of components, fabrication, and formats that apply in a complex matrix, such as a plant sample. Although the sensitivity of the LFA method is incomparable with that of the ELISA-based method or new-generation chromatographic methods, it is a representative analytical method suitable for the rapid screening of plant samples. The cost-effectiveness, selectivity, and simplicity of this method are exceptional. However, the main developmental obstacle is the specific antibody production for a particular antigen because the selectivity cannot be improved once a system is developed. There is room for studies to investigate sensitivity using the enzyme-based LFA or fluorescent probe, as this could cause a big leap in improvement. Furthermore, the scarcity of colloidal gold nanoparticles during the pandemic could lead to a stock shortage. The invention of ecofriendly alternative nanoparticles that exhibit identical or superior sensitivity is challenging. Thus, studies on LFAs for phytoproduct analysis remain evergreen.
